# Medical News and Miscellany

**Published:** 1895-01

**Authors:** 


### Medical News and Miscellany.

**Dr. A. Jay Sibley** has removed from West Point to Austin, Texas.

**The Life and Labors of Pasteur,** by the late Prof. Charcot, is published in the *Cosmopolitan* for January. It is intensely interesting to doctors.

**Dr. W. E. Shelton,** of Austin, sustained a serious loss on the night of December 22d, having his office and library badly damaged by fire and water.

**Death of Dr. Perl.**—The JOURNAL is pained to announce the death of Dr. M. Perl, of Houston, a prominent long-time physician of the Bayou city. He died of apoplexy, January 2d, inst.

**Dr. Van B. Thornton** has removed from Galveston back to Hempstead, his former home. The change is necessitated by rheumatism; the climate of Galveston did not agree with him. The Thornton Infirmary, at Hempstead, was burned on the 30th of November. It will be rebuilt.

**Save 50 Cents.**—The Mississippi *Medical Monthly*, one of the best and most spirited of our Southern exchanges, can be had for fifty cents by subscribing for it through us. The TEXAS MEDICAL JOURNAL and the *Mississippi Medical Monthly* club, both for $2.50. Here is a good opportunity to get two of the best Southern journal for a very small sum.

**Prof. Jas. Kennedy, M. D., Ph. G.,** Professor of Chemistry in the Medical Department, University of Texas, and President of the School of Pharmacy, has resigned his position, and will return to San Antonio and resume practice, his resignation to take effect at the end of the present session, June 1st. So far as we know, no one has been selected to fill the chairs.

**The** death rate of Chicago, according to official report of the City Bureau of Vital Statistics, is as follows: Deaths in November, 1894, 1,633; population, 1,600,000. Ratio in 1.000 inhabitants, 1.02. Annual deaths per 1.000, 12.25. An excellent showing for Chicago. It is the result of sanitation, and is about one half the death rate prior to digging the big ditch.

---

**Medical Consulation Book.**—This great work, by Dr. Hachenberg, which has received very high commendations at the hands of reviewers, and which was sold at $7.50 per volume, can now be had in club with the JOURNAL for $2.50,—the two for $4.50,—by addressing this office. By a special arrangement with the publisher, we are enabled to offer the few remaining unsold copies of this splendid work at just 33⅓ cents on the dollar, to close them out. This is the original revised and unabridged work. Here is a rare chance, doctor.

---

**Doctor,** do you need a battery? The JOURNAL has several new McIntosh batteries, both Galvanic and Faradic, which will be sold to subscribers at *less than manufacturer's best discount prices*. We have one 24-cell Galvanic, the catalogue price of which is $55; one 12-cell ditto, the catalogue price $30; one McIntosh "No. 3 Physician's Battery," $30, and a ditto "Family Battery" listed at $10. From the above list prices we will make a *large deduction*. We solicit correspondence. If you want one of the above, we will make the price satisfactory.

---

**The death rate** of New Orleans for the last week in December, 1894, according to official report of the State Board of Health, was as follows: "Death rate per 1,000 per annum for the week —Whites, 35.35; colored, 59.31; total white and colored, 41.91." That indicates a terrible mortality. The principal diseases causing these deaths (204 for the week, in estimated population of 254,000) were: Typhoid fever, 5; phthisis pulmonalis, 17; malaria, 5; heart disease, 8; bronchitis, 6; pheumonia, 19; chronic Bright's disease, 10; infantile "debility," 13, etc.

---

**Testimonial to Sir Joseph Lister.**—It is proposed to raise a subscription of 2 guineas each (about $10.23) with which to pay for a portrait of Sir Joseph Lister, to be presented to him as a testimonial. He has recently retired from active hospital and teaching work, and this is thought by his friends and admirers to be a fitting occasion to thus testify their regard. So we are informed by circular letter from J. Fred W. Silk, Honorary Secretary. Those who wish to subscribe, should address Mr. Silk, 39 Weymouth St., Portland Place, London W., England.

**Changes in the Memphis Medical College.**—In consequence of the death of Prof. Sim, the following changes have been made in the faculty organization of the Memphis Medical College: Prof. W. B. Rogers, M. D., becomes Dean, and Prof. B. G. Henning takes the chair of Principles and Practice of Medicine, while Prof. H. D. Wilford succeeds Dr. Henning in the chair of Materia Medica and Therapeutics. Prof. A. G. Sinclair, M. D., succeeds Prof. Sim as editor of the *Memphis Medical Monthly*. The school and the journal have both lost their strongest pillar in the death of Dr. Sim.

---

**An oculist,** satisfactorily endorsed as to ability and character—none other need apply—can, for $2,500, half cash, balance to suit, if secured, buy, out and out, a well established special practice paying now, and for several years past, from $500 to $700 cash *per month*, in one of the best cities in Texas. Seller will remain long enough to *thoroughly establish his successor; will guarantee delivery*, and then retire altogether from special practice in Texas. Handsome suite of offices, rent free for a term of years, with complete equipment of furniture, fixtures, instruments and appliances, go with the practice. A rare offer. Double the amount has been refused for a limited partnership. Satisfactory reasons for selling. Purchase of residence optional. Address "Oculist," care TEXAS MEDICAL JOURNAL, Austin, Texas.

---

**Antitoxine in America.**—Supervising Surgeon General Wyman, M. H. S., announces (abs. San. Reports, Dec. 14, 1893), that Passed Assistant Surgeon J. J. Kinyoun has returned from Paris and Berlin, where he thoroughly informed himself about antitoxine, its mode of preparation and use; and invites boards of health to detail medical men to go on to Washington and study the new treatment under Dr. Kinyoun.

Texas should, by all means, take a hand in this. Texas should have a laboratory for the preparation of the immunizing serum, and the new therapy should be taught. Something of the kind could best be established in connection with the Medical College, which is endowed and its officers paid by the State. There surely must be a fund available for such work, out of the Medical College appropriation. There is none available out of the Quarantine fund, and it would be difficult, we assume, to get the legislature to see the necessity of a special one. In Texas horses are abundant and cheap; ponies, suitable for the purpose of inoculating, can be bought at, from five to fifteen dollars each. Whc will move in this important matter?

---

**The Deadly Oyster.**—An epidemic of typhoid fever at Middletown, Conn., has been traced to eating raw oysters, the last thing in the world to be suspected of harboring "germs." It was learned that the dealers were in the habit of depositing their stock of oysters in the mouth of a little river, and that a private sewer discharged its contents just above the bed; further, that the excreta of a typhoid fever case had been passed through the sewer; hence the contamination.

Thus the necessity of government control of all streams, and prevention of contamination, becomes more imperative day by day. We do not see why it is that the same precautions are not taken with typhoid fever as with cholera, diphtheria, scarlet fever and small-pox. It is a disease dangerous to public health; the poison is known to be in the dejecta, and it is known also that a person must swallow that poison in order to get typhoid fever. It would seem almost criminal, therefore, that through neglect of nurses and others in charge of a case, innocent persons should be subjected to the possibility of swallowing this poison. To throw the dejecta of typhoid fever patients where it can get into running water, should be made an indictable offense. Why do not physicians impress upon families the necessity of destroying this poison, as they do with regard to the dejecta of cholera, and the sputa of consumptives, diphtheria, scarlet fever, etc.?

---

**Medical Legislation.**—It will be seen by reference to Dr. Downs' letter that the Eclectics are moving in the direction of medical legislation and propose co-operation. Well, for our part we want no co-operation either with the Eclectics or the Homeopaths. If we can not get legislation to suppress quackery without having to recognize Eclectics and Homeopaths, we will do without it. The JOURNAL refuses to recognize any "schools;" there is but one school, the regular physician, and as those irregulars have always opposed every attempt on the part of the regular profession to secure suitable legislation, we will certainly not consent to join hands with them against all *other* irregulars. If we co-operate with one class, where shall the line be drawn? We let down the bars to admit one kind of stock and all the sheep and goats will get in. If the medical profession accept the assistance of Eclectics and Homeopaths in the matter of legislating against quackery, we should call on the Christian Scientists and all the rest for help.

No; let's go it alone, hit or miss, succeed or fail. We can not compromise our dignity as physicians by giving even this quasi recognition to these irregulars.

And, indeed, if we understand the matter as proposed by Dr. Wooten, it is to ask for a law that will recognize no diploma except from a high grade school; that does away with all "boards" and requires no examinations,—the only qualification being the possession of a diploma from a college which requires not less than three courses of not less than six months each, in three (or more) separate years; said list of colleges to be prepared by a *commission* of regular physicians. In said event we do not need, nor want, nor will we accept "assistance" from these parties.

---

**Ring Out the Old Year; Ring in the New.**—The "Red Back" sends greeting to its hosts of readers, and wishes them all a happy New Year. We wish them a big improvement on old 1894, which knocked out so many, and slightly damaged all the others, and bid them cheer up; take a fresh hold on life, and determine to get more good out of 1895 than most of us did out of 1894. It was a hard year on the natives sure enough; but still, we have much to be thankful for. The "Red Back" has felt the universal depression, of course, but we are still in the ring, undismayed—not at all discouraged; but on the contrary as full of hope, vim, vigor and determination as in its younger days when it was "but a little kid." Our friends have shared with us and have stuck to us; and thus encouraged we are still determined to give them *the best medical journal* that can be gotten out in Texas, or anywhere else.

We are glad to see that organization is still going on, and in a little while we hope to see the profession fully organized into local societies. *Then* we can have a yearly convention of medical delegates, even though the State Medical Association should continue the even tenor of its way, and decide to not make any change.

APROPOS of the above, we are informed that the seeds sown by the JOURNAL some months since have, some of them, fallen in good ground and have sprouted. Our editorial "Shall the State Association be abolished?" in which we advocated a change in its organization, has attracted the serious attention of the thinking members, and they realize that something must be done to check the downward course of the Association, or its days are numbered; and correspondence has been going on amongst the leaders, which we are informed will result in bringing about an active consideration of the subject at our Dallas meeting.

We also learn that steps are being taken to secure a full attendance at Dallas. We shall live in hopes.

---

**Vital Statistics.**—It is much to be regretted that in Texas there is no provision for the collection and preservation of vital statistics. Every effort on the part of the medical profession to secure such legislation as would make it a duty to report and record every birth and every death, has been a failure, because the legislators do not appreciate the importance, nay, the necessity, of such record. True, in the office of Commissioner of Insurance, Statistics and History, there is a pretense of collecting such statistics, but as it is not made compulsory on physicians and midwives to report births and deaths, it is not done, only just "when they feel like it," we suppose, for, in a report of that department awhile ago, about one-fourth of the deaths were put down as "cause unknown." As the Texas Legislature is now in session, and the JOURNAL understands that an effort is to be made to secure legislation to "regulate the practice," we suggest the following as timely, and that the movers in the effort at legislation incorporate in their bill some provision for the registration of births and deaths.

Dr. Arthur R. Reynolds, Health Commissioner of Chicago, in a paper on "Needed Legislation," which was read before the State Board of Health Auxiliary Sanitary Association, in Springfield, Illinois, in November, 1894, says:

Under our system of deputizing this duty to the county clerks, without even a nominal compensation to the reporting physician or midwife, there is possibility of immediate oversight, and the chances for defects in the records are multiplied. There is no doubt that births go unreported to a very large extent in all parts of the State, and this is also true as to deaths, except in the cities and towns which enforce the burial permit ordinance of the State Board of Health, or its equivalent.

The advantages and needs of accurate birth registration are daily demonstrated in the experience of the health department of the city of Chicago; questions are constantly arising which cannot always be answered, for the lack of it. This is not a matter of merely scientific interest; it is a practical one, and I think it should be impressed upon our legislators that what we ask in this direction is not solely in the interest of State medicine, or of sanitary administration. As a matter of fact, while these interests are of importance to us, the full and authentic record of births is of greater and growing importance to the public.

There is hardly a relation in life, from the cradle to the grave, in which such a record may not prove to be of the greatest value. For example, in the matters of descent; in the relations of guardians and wards; in the disabilities of minors; in the administration of estates; the settlement of insurance and pensions; the requirements of foreign countries in matters of residence, marriage and legacies; in marriage in our own country; in voting and in jury and militia service; in the right of admission and practice in the professions and to many public offices; in the enforcement of laws relating to education and to child labor, as well as to various matters in the criminal code; the irresponsibility of children under ten for crime or misdemeanor; the determination of the "age of consent," etc. As the country becomes more densely settled, and the struggle for existence sharper, many of these matters, which have hitherto been of minor significance, will take on a deeper meaning and acquire greater importance.

---

**Contagious Diseases.**—The State Health Officer has just issued the following circular letter to county physicians in Texas:

DEAR DOCTOR:—I respectfully invite your careful consideration of the quarantine law passed by the twenty-second legislature, wherein your official duties are clearly defined, and the fact made apparent that the responsible duties imposed upon you can not be borne by any other person.

When a case of small-pox, diphtheria or scarlet fever occurs in your jurisdiction, promptly notify your county judge and your commissioners, and ask for the authority to establish quarantine as directed by law. Even one day's delay might multiply the number of those exposed to the infection, and greatly enlarge the circle of danger.

When the report of a case of acute infectious disease reaches you, proceed at once to investigate; and if in doubt as to a diagnosis, give public health the benefit of the doubt, and assume that it is dangerous until time demonstrates the fact that it is not dangerous. If it is not an infectious disease no one is hurt, and sensible people will not object to the inconvenience you have given them. If the report proves to be correct, place a yellow or a red flag in some conspicuous place about the front premises, separate the one sick from the well, and if possible to do so, in case of small-pox, vaccinate all who have been exposed. This isolation and segregation should be enforced for at least ten days after the dismissal of the last small-pox patient.

With regard to either of these diseases, no definite time can be fixed when a patient may safely go out. As a general thing, when the patient is fully convalescent, and all desquamation has ceased, he should be cleansed thoroughly with a warm bath and soap for three or four consecutive days. If at the end of that time there is no roughness of skin, he may be dressed in clean clothes and taken from the room; he is no longer, then, a source of infection. All rags and articles of little or no value that have been used, or allowed to remain in the sickroom, should be burned. All sheets, towels, pillow-slips, spreads, in fact, all articles that will bear it without injury, should be thoroughly boiled. The room and furniture should be cleansed and disinfected. The ceilings and walls, if of ordinary hard finish, should be scraped and whitewashed or calsomined; ordinary plank rooms may be whitewashed. All woodwork should be rubbed off with damp cloths, and the floors well scrubbed. Care should be taken to remove all dust from the ledges of the doors and windows, and all cloths used in the cleaning process should be burned. Upholstered furniture, or articles too valuable to be burned, or which can not be scrubbed or boiled, can be disinfected in situ. Cracks or openings of all kind in the room should be stopped and the room thoroughly fumigated with burning sulphur. Pillows, mattresses and feather beds, all hair stuffed cushions or furniture should be ripped open, so as to subject their contents to the action of the sulphurous fumes.

The above is, in a general way, about the best course to pursue, modified, of course, to suit circumstances and individual cases. Too much care can not be taken to prevent contagion.

Your guards ought always to be reliable men, who will minutely carry out your instructions, and they should never be allowed to enter infected houses. Nurses should be impressed with the absolute importance of keeping at a safe distance from the guards and all other persons liable to contagion.

The physician in charge should wear a long rubber coat when within the infected house, and immediately after leaving it, should wash his coat, hands and face in some strong germicidal solution,—the bi-chloride I think preferable,—and brush hat, boots and shoes with a whisk-broom dipped in the same solution. These precautions do not give a partial or possible immunity to the physician, but they do protect others, and give assurance to intelligent communities that their medical officer is not a walking nidus of infection.

The State Health Officer will at any time when necessary, visit cases of doubtful diagnosis, and confer with county or city health officials. Whenever any community is threatened with an invasion of contagious or infectious disease, a fearless performance of duty by local health officers, will, in the majority of cases, avert an epidemic. The public look to them for protection, and no sacrifice of self, or of comfort or convenience, should be spared to secure it.

I have the honor to be, your obedient servant,

R. M. SWEARINGEN, M. D.,
State Health Officer.

copy be sent to the family of the deceased, also be published in the *Memphis Medical Monthly* and in the daily press.

The TEXAS MEDICAL JOURNAL deeply deplores the loss to the profession of this great and good man: we mourn his loss, too, as that of a personal and very dear friend; and we fully share in the sentiments above so beautifully expressed. Peace to his ashes. Dear, generous, warm-hearted Sim! The poor of Memphis are called on to mourn a loss, sad indeed to them,—a benefactor of his race.

